# Informal carer involvement in the transition of medicines-related care for patients moving from hospital to home: a realist review protocol

**DOI:** 10.1136/bmjopen-2024-091005

**Published:** 2024-09-11

**Authors:** Matthew Cooper, Olivia Atkinson, David Black, Laura Lindsey, Christina Cooper, Hamde Nazar, Geoff Wong, Carmel Hughes, Charlotte L Richardson

**Affiliations:** 1 Newcastle Patient Safety Research Collaboration, Newcastle University, Newcastle upon Tyne, UK; 2 School of Pharmacy, Newcastle University, Newcastle upon Tyne, UK; 3 Social Work, Education and Community Wellbeing, Northumbria University, Newcastle upon Tyne, UK; 4 Nuffield Department of Primary Care Health Sciences, Oxford University, Oxford, UK; 5 School of Pharmacy, Queens University Belfast, Belfast, UK

**Keywords:** Caregivers, Caregiver Burden, Medicine, Hospital to Home Transition

## Abstract

**ABSTRACT:**

**Introduction:**

Transition of care for a patient between hospital and home can cause disruption to normal routines, increasing the risk of medicines-related harm. The transition from hospital to home is more complex when a patient does not self-manage their medicines but relies on an informal or unpaid carer (eg, spouse, family member or friend) to provide support. Given the day-to-day medicines-related support provided by informal carers, there is a need to understand how informal carers manage the transition of care from hospital to home; what aspects of hospital discharge act as barriers and facilitators to their involvement and when, how and why these impact patients.

**Methods and analysis:**

A realist review will be undertaken to develop a programme theory. The programme theory will theorise which medicines-related interventions are useful to carers, and how they are useful. It will outline what aspects of those interventions are the most useful and why, and how context influences engagement and medicine-related outcomes. The review will be reported in line with the Realist and Meta-narrative Evidence Syntheses: Evolving Standards guidelines. Data will be selected, screened and extracted based on defined inclusion and exclusion criteria and relevance to the developing programme theory with the involvement of at least two authors acting independently. Inclusion criteria relate to the relevance to hospital discharge where patients move back to their home, where a carer is involved and where interventions relate to medicines use. Searches will be conducted in PubMed, CINAHL (via EBSCOhost) and EMBASE databases (see supplementary materials for a draft search strategy).

Patients and public, participation, involvement and engagement (PPIE) will be incorporated into all stages of the review through iterative engagement and discussion with patient, carers and representatives from carer organisations. The review will follow four steps: (1) development of the initial programme theory, (2) evidence search, (3) selection, extracting, and organising data and (4) synthesising evidence and drawing conclusions.

Informal carer involvement in transitions of care is a complex and varied phenomena. The programme theory will be shaped by sustained PPIE reflecting the priorities and experiences of lived experience. The realist review be progressively focused so we can develop a better understanding of carer involvement in patient transitions when moving from hospital to home relating to medicines use.

**Ethics and dissemination:**

Ethical approval is not required. The findings of the review will be disseminated via journal articles and through patient and public facing resources such as a visual patient–public-carer focused summary.

**PROSPERO registration number:**

CRD42021262827.

**STRENGTHS AND LIMITATIONS OF THIS STUDY**

- This protocol reports on a planned realist review using the Realist and Meta-narrative Evidence Syntheses: Evolving Standards to ensure transparency and rigour.
- In line with realist methods, patients and public, participation, involvement and engagement is established throughout the proposed review to ensure the work remains grounded in the lived experiences of patients, carers and the public.
- The realist review is registered with PROSPERO CRD42021262827.

## Introduction

 Transitions of care for a patient between hospital and home can cause a significant degree of disruption to their normal routines, including to patient’s medicines and healthcare management.^1^ Medicines-related problems are reported to occur in 20% of patients at discharge from hospital, of which 60% are preventable.^2^ These typically stem from poor communication and/or discontinuity in care coordination and inadequate explanations about medicines, leading to medicine omissions or errors and patient/carer anxiety or confusion at discharge.^3^

Medicines-related harm (MRH) during the transition from hospital to home is well recognised and adds to the UK’s National Health Service (NHS) costs, detrimentally impacts quality of life and presents a challenge to overstretched NHS resources.^1^ Within 30 days of hospital discharge, 17%–51% of older people experience MRH.^4^ In the UK, 28% of adults ≥65 years are believed to use health services (both primary and secondary) due to MRH within 8 weeks of discharge, costing ca. £400 million annually.^5^

Issues relating to medicines changes following a hospital stay are more complex when a patient does not self-manage their medicines but relies on an informal and unpaid caregiver (eg, a spouse, family member or friend) to provide support. An informal caregiver is defined as:

Anyone who looks after a family member, partner or friend who needs help because of their illness, frailty, disability, a mental health problem or an addiction and cannot cope without their support. The care they give is unpaid.^6^

This definition is adopted for the proposed review, but the term ‘carer’ is used synonymously for the remainder of this protocol.

Many medicines management activities are undertaken by carers. Research about their involvement in transitions of care from hospital to home focuses on a range of contexts (eg, different disease states, diagnoses and intervention location and timing, etc) and carer roles (family, friends, community members, etc).^7 8^ Research exploring carer experiences in supporting medicines use for patients with dementia described three areas of concern: (a) inadequate information about medicines management; (b) limited caregiver engagement in medicine-related decisions and (c) difficulties relating to medicine supply post-discharge.^9^

There is some evidence that carer involvement in transition of care pathways at hospital discharge can improve patient outcomes, but carer involvement is far from embedded within usual practice.^3 10 11^ The nationally commissioned Discharge Medicine Service in the UK recommends carer involvement in the service without specifying the operational details about who, how or when to involve carers in the discharge pathway.^12^ Due to the complexity and variation inherent in such transitions of care pathways, it is not clear how carers can best be involved in supporting medicines management and transition from hospital to home, in which contexts, for which patient groups and for what benefits to the patient.^11^ These complex interventions may be multifaceted, open to external influences and subject to change.^13^ Therefore, to better understand the complex relationships between patients, their carers and medicine management when patients are transitioned out of hospital, we will undertake a realist review.^13 14^

## Methods and analysis

### Aim

This review aims to establish the role of carer involvement in transitions of care from hospital to home in relation to the management of medicines. How carers provide support, to what extent they support and under what circumstances are carers able to provide support towards patient care in relation to medicines management.

To address the aim, the review uses the Context, Intervention, Mechanism, Outcome framework,^15^ to answer four questions:

1. Context: what are the contexts in which carer involvement in transitions of care and medicines management support patient outcomes?
2. Intervention: what are the details of a medicines-related intervention that includes carers and is provided to help transition patients from hospital back to their homes as described in the literature?
3. Mechanism: what are the context-specific mechanisms which are likely to contribute to these benefits?
4. Outcome: what are the outcomes, related to the context and mechanism, achieved by the support from carers in the transitions of care relating to medicines management, for people who have been discharged from hospital?

### Rationale

The realist approach to evidence synthesis lends itself to investigate complex problems or challenges. This approach will help to understand which and how medicines management strategies used during transitions from hospital to home are useful to carers, what aspects are most useful and why, how contextual factors influence engagement with these barriers or facilitators and what outcomes arise.

Research on the role of carers in the transition from hospital to home for older patients, is largely qualitative, describing the carer and/or patient perspective to identify barriers and facilitators. A recent systematic review found that carers described communication about medicines when patients were being transferred as haphazard and disorganised, lacking shared decision-making.^11^ The authors recommended strategies such as family meetings, clinical bedside handovers, ward rounds and admission and discharge consultations to aid carer involvement. The findings from this review, and several other seed papers, will inform our initial programme theory in line with a realist approach.^13^

### Patient and public involvement

Patients and public, participation, involvement and engagement (PPIE) are a key element of a realist review.^16^ PPIE and stakeholder engagement will advise on all stages of the project, including the development of programme theory, giving feedback and advice on emerging findings, output development and dissemination through iterative engagement and discussion. Members of the group will include patients and carers with lived experience of hospital discharge. Stakeholders are organisations and groups who work to support carers, promote PPIE and tackle health inequalities. Specific organisations we are working with include: ConnectedVoice HAREF (ConnectedVoice health equality for ethnically minoritised communities), Newcastle Carers and Voice-global.org.

To ensure sustained and meaningful PPIE, we have a member of the research team who has lived experience of the burden of the discharge process as a carer (DB). DB as a carer has relationships with national and regional patient and carer organisations and, in addition to providing his own lived experience, will identify key stakeholders.

### Study methods

This protocol is being reported in accordance with the reporting guidance provided in the Preferred Reporting Items for Systematic Reviews and Meta-Analyses Protocols (PRISMA-P) statement (see online supplemental materials).^17 18^ The realist approach will follow the Realist and Meta-narrative Evidence Syntheses: Evolving Standards guidelines (RAMESES) quality standards to ensure transparency and rigour^13^ and will follow the processes set out by Pawson.^19^ Ethical approval is not required. The review is expected to run from March 2024 to March 2025.

**Figure 1:**
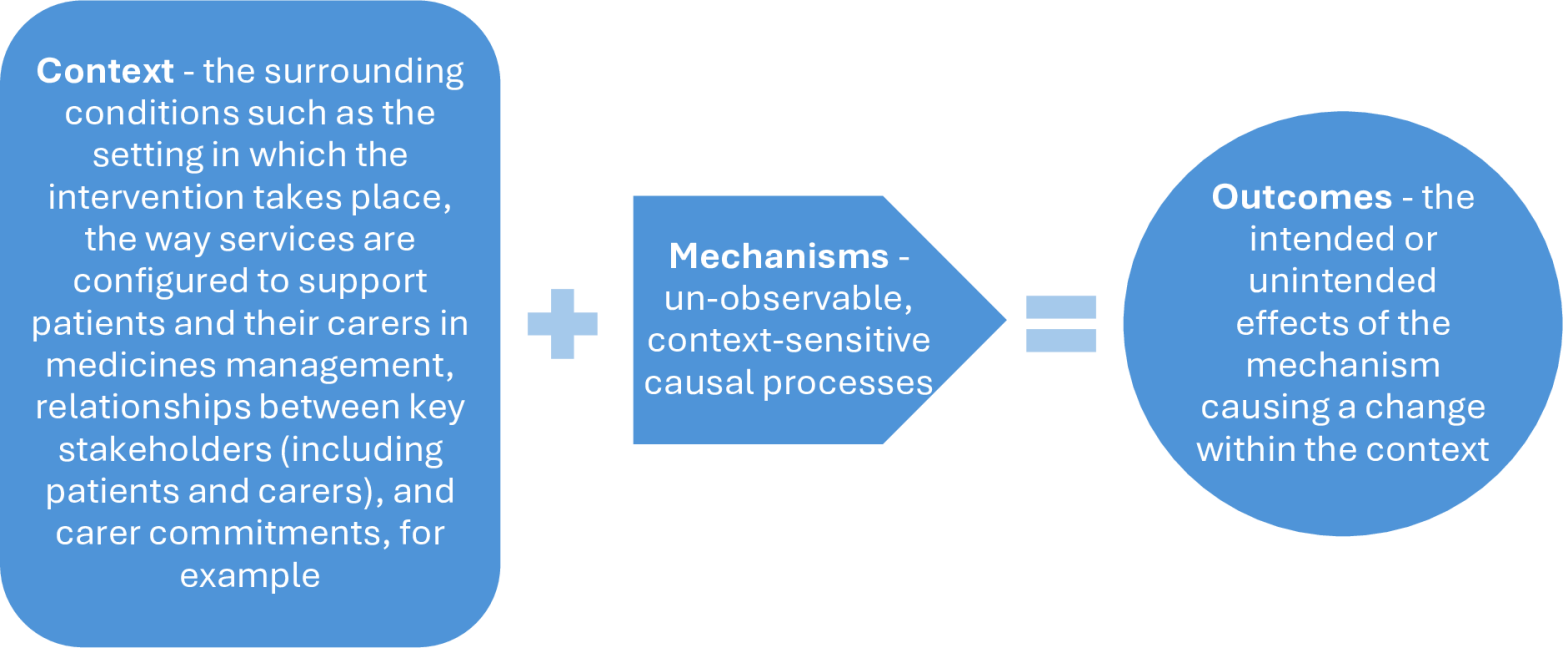
Context+Mechanism = Outcome configurations (adapted from Dalkin *et al*)*.*^23^

A realist review approach acknowledges that interventions may have different effects for different people, depending on the context of an intervention. This understanding as captured in the heuristic formula Context (C)+Mechanism (M)=Outcome (C+M=O) (also expressed as C+M=O; CMOs) (see figure 1). This heuristic explains the causation for an outcome for any complex intervention—namely something that functions as context triggers a mechanism, which in turn causes an outcome. To explain the outcomes within a complex intervention, multiple configurations of CMOs (CMOCs) may be needed.

#### Step 1: development of the initial programme theory

A realist synthesis begins by defining the scope of the review to inform the development of an initial programme theory. The initial programme theory will build on the authors’ understanding of the relevant literature, identify literature that explores interventions which consider patients and carers for different groups (older people, those with disabilities dementia, mental health, etc), carer groups (types of family members, friends, etc), and which discuss outcomes for both patients and/or carers.

We will undertake pilot scoping searches to ensure the relevancy of possible terms identified from known literature on the topic and clinical experience using PubMed as a testing database. Terms are related to medicines management and transitions of care from hospital to home that include carers to help us understand what is already available, situating our research within this. The pilot scoping searches will provide a conceptual map of relevant information to inform the initial programme theory (eg, relevant theory, potential mechanisms and pathway data). This will also inform discussions with stakeholder and PPIE representatives to consider scope, collate potential areas for searching or specific search terms and facilitate the development of the initial programme theory. The initial programme theory will also inform development of the search terms for step 2.

#### Step 2: evidence search

Using the terms identified in step 1 and in consultation with a specialist librarian, systematic identification of evidence to refine the initial programme theory will be developed. Literature searching will be conducted in PubMed, CINAHL (via EBSCOhost) and EMBASE databases (from inception until the point the search is conducted). In addition, we will use search strategies such as citation tracking, kinship and sibling papers and snowballing to help identify all data relevant to the programme theory. Additional searching may be undertaken as required to contribute to further refinement of the programme theory and/or development of CMOCs, and until we have obtained sufficient data to test (confirm, refine or refute) the programme theory, this may include grey literature.

#### Step 3: selection, extracting and organising data

Documents identified in step 2 will be selected for inclusion using a three-step screening process. First, we will screen all potentially relevant documents retrieved by the search by title and abstract, and following that, full text, against inclusion and exclusion criteria (see table 1).

**Table 1:** Inclusion and exclusion criteria for the screening of documents to be included within the realist review

Inclusion criteria	Exclusion criteria
Patients who were discharged from hospital to home who have a carer involved in their care Interventions that are medicines-related and aim to improve the transition of care between hospital and home	No carer involvement in patient care Non-medicines-related interventions Any studies with paediatric patients

The term ‘carer’ includes any form of informal carer, for example, a family member or a friend.

Second, those that meet the inclusion criteria will be read in detail and our final decision on inclusion in the review will be based on the criteria of relevance and rigour as described by Pawson.^19^ To ensure consistency in the application of the inclusion criteria, a second team member will use Rayyan software to blindly screen a 10% random sample of documents in duplicate. Any discrepancies will be resolved through wider team discussion.

We will use established quality appraisal tools to judge the rigour of data in included documents. We will do this when a document contributes a substantial amount of data which results in a change to the programme theory. Hence, it is important for us to be able to trust these data by assessing the rigour of the methods used to generate them. Where there is uncertainty as to how to judge rigour, we will predominantly consider the relevance of data to the programme theory. We will take this approach as even data that are of questionable quality may still provide relevant information to inform programme theory development.^20^

We will judge the explanatory plausibility of the programme theory using the criteria of consilience, simplicity and analogy.^21^ Despite these measures, threats to the plausibility of the programme may still occur, particularly if sections of it are based predominantly on data that we would judge (globally) to be of questionable rigour. We would highlight where this is the case in publications as a limitation.

Data from relevant full body text-documents will be extracted using a suitably designed and piloted standard data collection process. Key characteristics of each included document will be extracted into an Excel spreadsheet, and the full-text documents will be uploaded to NVivo so relevant data can be organised and coded. Coding will involve extracting relevant sections of text according to how these data can contribute to programme theory development.

#### Step 4: synthesising evidence and drawing conclusions

**Figure 2:**
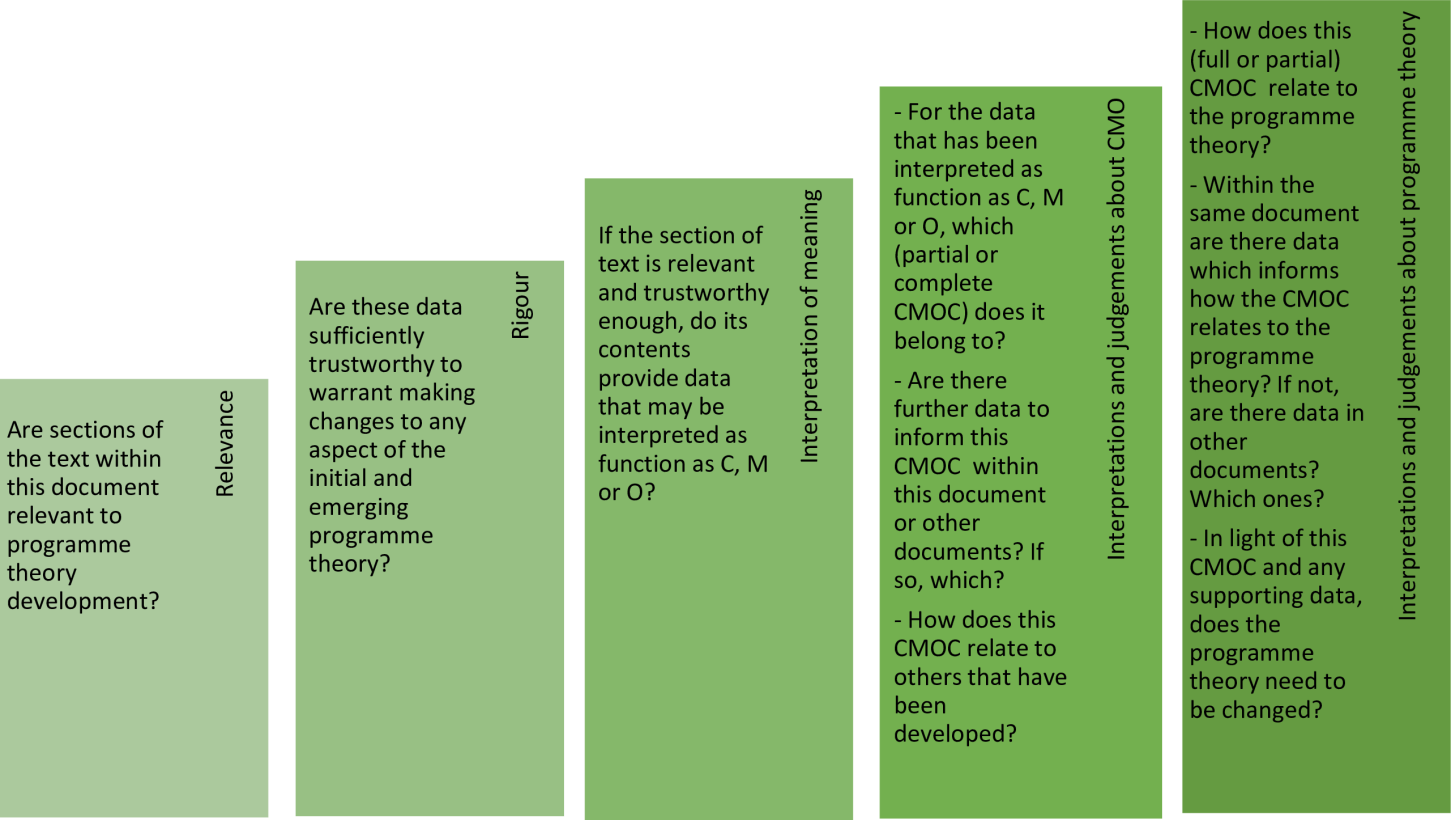
Analysis process of determining the relevance and rigour of the sources used to inform the programme theory.^13 24^

Data analysis will involve the use of a realist logic of analysis with the goal of using the data from the included documents, to further refine the initial programme theory developed in step 3. Data coding will be deductive (informed by initial programme theory), inductive (coming from the data within included documents) and retroductive (inferences are made based on interpretations of the data within included documents about underlying causal processes—ie, mechanisms). We will use a series of questions about the relevance and rigour of content within documents as part of our process of analysis (see figure 2).

Data to inform our interpretation of the relationship between CMOCs will be sought not just within the same source, but across sources (eg, mechanisms inferred from one document could help explain the way contexts influenced outcomes for some interventions but not others). Synthesising data from different documents are often necessary to compile CMOCs, since the data to inform all parts of the configurations may not be found in the same documents.

During the review, we will move iteratively between the analysis of particular examples, refinement of programme theory and further iterative data searching to test particular elements of the theory. We will have regular meetings with our PPIE and stakeholder representatives to discuss the literature, and sense check the developing programme theory. The programme theory and a summary of the literature will be discussed with these groups who will be asked to comment on its resonance with their perspectives and lived experience. We will address any gaps in the theory that persist (eg, through additional literature searches). Discussions with the PPIE and stakeholder group will identify elements which will be best taken forwards for evaluation through further research.

## Discussion

Carer involvement in medicines management is a complex and varied phenomena and as such there are limitations on how much can be captured in a single realist review. For this reason, the review will focus on aspects of carer involvement in hospital discharge relating to medicines management deemed to be most important by the research team, and PPIE and stakeholder group.^19^ As such, the final programme theory will only capture a proportion of a complex issue. However, further research will be able to test and hone the programme theory to establish a more comprehensive theory of carer involvement in patient transitions from hospital to home relating to medicines management.

To date, PPIE representatives (patients, carers and members of the public) and stakeholder organisations have provided feedback on the research questions, study methods and the proposed PPIE. These discussions identified three observations:

1. Unclear messages regarding medicines management at discharge have a significant impact on patient care.
2. Unique relationships between the carer and patient are scarcely acknowledged and discussed by professionals.
3. Carer and patient health and well-being are inextricably linked, with carer health potentially suffering due to the burden of caring for someone else. This is worsened at transitions of care.

These discussions have shaped our protocol as follows:

- The importance of inclusion of a diverse patient and carer voices in the review. Through ConnectedVoice HAREF, we will aim to engage with ethnically minoritised communities and ensure the PPIE and stakeholder activities are inclusive.
- Considerations as to how to best include individual carers in the review through flexibility around caring commitments, appropriate renumeration which include respite care provisions to allow carers to participate (in line with UK National Institute for Health and Care Research recommendations).^22^
- Advice on dissemination and accessibility of the final programme theory, such as the production of a patient–public-carer focused summary.

This realist review aims to take a broad view, situated in lived experiences of patients and carers, when considering the question of carer involvement in transition from hospital to home and medicines management. CMOCs will incorporate outcomes relating to both patients or carers given the complex and inter-related nature of carer–patient relationships. The findings are planned to inform the future development and testing of an intervention or model of care related to medicines management that most appropriately, safely and effectively involves carers in hospital to home discharge care for the benefit of the patient.

## supplementary material

10.1136/bmjopen-2024-091005 online supplemental file 1
