# Supplementary material for: Informal carer involvement in the transition of medicines-related care for patients moving from hospital to home: a realist review protocol
Source: BMJ Open. 2024 Sep 11;14(9):e091005. doi: 10.1136/bmjopen-2024-091005 (PMC11409271; doi:10.1136/bmjopen-2024-091005)
Supplement: online supplemental file 1 [file bmjopen-14-9-s001.pdf]

Supplementary materials - Informal carer involvement in the transition of medicines-related care for patients moving from hospital to home: a realist review protocol

Draft search strategy

Transition:

- transition points
- transition of care
- transition*
- transfer
- discharge*
- admission*

Medication:

- medication management
- medicines management
- medicines*
- medication*

Caregiver:

- caregivers
- carers
- relatives
- family
