# Supplementary material for: Informal carer involvement in the transition of medicines-related care for patients moving from hospital to home: a realist review protocol
Source: BMJ Open. 2024 Sep 11;14(9):e091005. doi: 10.1136/bmjopen-2024-091005 (PMC11409271; doi:10.1136/bmjopen-2024-091005)
Supplement: Supplementary file 2 [file bmjopen-2024-091005.reviewer_comments.pdf]

PEER REVIEW HISTORY

BMJ Open publishes all reviews undertaken for accepted manuscripts. Reviewers are asked to complete a checklist review form (**http://bmjopen.bmj.com/site/about/resources/checklist.pdf**) and are provided with free text boxes to elaborate on their assessment. These free text comments are reproduced below.

ARTICLE DETAILS

**TITLE (PROVISIONAL)**	Informal carer involvement in the transition of medicines-related care for patients moving from hospital to home: a realist review protocol
**AUTHORS**	Cooper, Matthew; Atkinson, Olivia; Black, David; Lindsey, Laura; Cooper, Christina; Nazar, Hamde; Wong, Geoff; Hughes, Carmel; Richardson, Charlotte

This article was not externally reviewed at BMJ Open. Protocols that have been independently assessed prior to submission to BMJ Open are usually fast-tracked to publication on the grounds that further substantial changes will not be possible. This independent assessment will usually be external, independent review for both a substantial grant award from a non-commercial or government funder and ethics approval.
